# Human telomerase reverse transcriptase and glucose-regulated protein 78 increase the life span of articular chondrocytes and their repair potential

**DOI:** 10.1186/1471-2474-13-51

**Published:** 2012-04-02

**Authors:** Masato Sato, Kazuo Shin-ya, Jeong Ik Lee, Miya Ishihara, Toshihiro Nagai, Nagatoshi Kaneshiro, Genya Mitani, Hidetoshi Tahara, Joji Mochida

**Affiliations:** 1 Department of Orthopaedic Surgery, Surgical Science, Tokai University School of Medicine, 143 Shimokasuya, Isehara, Kanagawa 259-1193, Japan; 2 Biomedicinal Information Research Center, National Institute of Advanced Industrial Science and Technology (AIST), 2-42 Aomi, Koto-ku, Tokyo 135-0064, Japan; 3 Department of Biomedical Science & Technology, Institute of Biomedical Science & Technology (IBST), Konkuk University, 1 Hwang-dong, Gwangjin-gu, Seoul 143-701, Korea; 4 Department of Medical Engineering, National Defense Medical College, 3-2 Namiki, Tokorozawa, Saitama 359-8513, Japan; 5 Hiroshima University Graduate School of Biomedical Sciences, 1-2-3 Kasumi, Minami-ku, Hiroshima 734-8553, Japan

## Abstract

**Background:**

Like all mammalian cells, normal adult chondrocytes have a limited replicative life span, which decreases with age. To facilitate the therapeutic use of chondrocytes from older donors, a method is needed to prolong their life span.

**Methods:**

We transfected chondrocytes with hTERT or GRP78 and cultured them in a 3-dimensional atelocollagen honeycomb-shaped scaffold with a membrane seal. Then, we measured the amount of nuclear DNA and glycosaminoglycans (GAGs) and the expression level of type II collagen as markers of cell proliferation and extracellular matrix formation, respectively, in these cultures. In addition, we allografted this tissue-engineered cartilage into osteochondral defects in old rabbits to assess their repair activity in vivo.

**Results:**

Our results showed different degrees of differentiation in terms of GAG content between chondrocytes from old and young rabbits. Chondrocytes that were cotransfected with hTERT and GRP78 showed higher cellular proliferation and expression of type II collagen than those of nontransfected chondrocytes, regardless of the age of the cartilage donor. In addition, the in vitro growth rates of hTERT- or GRP78-transfected chondrocytes were higher than those of nontransfected chondrocytes, regardless of donor age. In vivo, the tissue-engineered cartilage implants exhibited strong repairing activity, maintained a chondrocyte-specific phenotype, and produced extracellular matrix components.

**Conclusions:**

Focal gene delivery to aged articular chondrocytes exhibited strong repairing activity and may be therapeutically useful for articular cartilage regeneration.

## Background

Osteoarthritis (OA), which is one of the most common, debilitating, and costly chronic disorders [1], is characterized by progressive degeneration or destruction of articular cartilage. Since the incidence of OA increases with age, the underlying mechanism of this disease may involve a loss of the capacity of chondrocytes to regenerate with age. In proliferative cells, telomeres from chromosomes gradually became shorter as a result of the DNA replication end problem. To prevent cessation of mitosis and premature cell death, telomerase is a ribonucleoprotein that is an enzyme which adds DNA sequence repeats (TTAGGG) to the 3' end of DNA strands in the telomere regions, which are found at the ends of chromosomes [2]. The telomerase allows for replacement of short bits of DNA known as telomeres, which are otherwise shortened when a cell divides via mitosis. In normal circumstances, without the presence of telomerase, if a cell divides recursively, at some point all the progeny will reach their Hayflick limit. With the presence of telomerase, each dividing cell can replace the lost bit of DNA, and any single cell can then divide unbounded. While this unbounded growth property has excited many researchers, caution is warranted in exploiting this property, as exactly this same unbounded growth is a crucial step in enabling cancerous growth. In immortal human tumor cells, the gene for the catalytic subunit of human telomerase reverse transcriptase (*hTERT*) is almost always derepressed [3]. Moreover, *hTERT *is not only an oncoprotein [2] but also a regulator of cellular differentiation [4,5].

As a result, immortalized human cells, such as epithelial and fibroblast cells [6] and chondrocytes [7,8], have been used as models of cellular aging. For example, Goldring [7] showed that primary human chondrocytes can be immortalized with retroviral transfection of 4 genes, including simian vacuolating virus 40 large T antigen and telomerase; however, stable transfection of *hTERT *in chondrocytes that are cultured in a monolayer allows maintenance of the proliferative capacity but not the chondrocyte phenotype. In contrast, Piera-Velazquez et al. [8] showed that exogenous expression of *hTERT *in chondrocytes that are cultured on polyhydroxyethylmethacrylate coated dishes increases their life span and maintains their chondrocyte phenotype. Thus, *hTERT *may extend the life span of chondrocytes.

Glucose-regulated protein 78 (GRP78) is a molecular chaperone in the endoplasmic reticulum (ER) that is induced by ER stress and prevents cell death as a result of homeostatic imbalance in the ER [9]. Although overexpression of *GRP78 *can limit the damage from ER stress in normal tissues and organs, the natural induction of GRP78 in neoplastic cells also may promote cancer progression and drug resistance [10]. Since *GRP78 *also is involved in the pathology of neurological diseases, such as Alzheimer's disease [11] and Parkinson's disease [12], GRP78 may have therapeutic cytoprotective effects to limit ER stress.

To determine whether *hTERT *and *GRP78 *can prolong the life span of chondrocytes and stimulate cartilage regeneration, we transfected rabbit articular chondrocytes with these genes and redifferentiated chondrocytes in a 3-dimensional atelocollagen honeycomb-shaped scaffold with a membrane seal (ACHMS scaffold) [13]. We used this type of scaffold because it is biodegradable, supports the growth of high-density cell cultures, and maintains the phenotype of articular chondrocytes [14-16]. In addition, to investigate the clinical relevance of our model to OA, we investigated whether the effects of gene transfection depend on the age of the cartilage by analyzing the proliferation, gross morphology, cellular content of DNA and proteoglycans, and gene expression level of type II collagen in the transfected chondrocytes.

## Methods

### Preparation of chondrocytes

All animal experiments in this study approved by Research Support and Intellectual Property of the University of Tokyo were performed in accordance with their institutional guidelines for the care and use of laboratory animals.

Chondrocytes were prepared as described previously [14]. Briefly, articular cartilage tissue specimens were collected from the knee and shoulder joints of 4 young male (4 weeks old, 1 kg) and 8 old female (4 years old, 4.5 kg) Japanese white rabbits (Tokyo Laboratory Animals Science Co., Ltd., Tokyo, Japan). Each rabbit specimen was soaked and stored separately in basal medium (BM) containing Dulbecco's modified Eagle's medium (DMEM)/F12 (Gibco; Invitrogen, Carlsbad, CA, USA) supplemented with 10% heat-inactivated fetal bovine serum (FBS) (Gibco), 50 μg·mL^-1 ^ascorbic acid (Wako Pure Chemical Industries, Osaka, Japan), and 1% Fungizone^® ^antibiotic-antimycotic solution (10,000 U·mL^-1 ^penicillin G, 10 mg·mL^-1 ^streptomycin sulfate, and 25 μg·mL^-1 ^amphotericin B; Gibco). When needed, cartilage samples were chopped into small pieces, and then digested for 1 h in DMEM/F12 containing 0.4% pronase E (Kaken Pharmaceutical, Tokyo, Japan), followed by digestion for 3 h at 37°C in DMEM/F12 containing 0.016% collagenase P (Roche Diagnostics, Mannheim, Germany). Subsequently, the digested samples were filtered with a cell strainer (BD Falcon™; BD Bioscience, Bedford, MA, USA) with a 100 μm pore size and the isolated cells were rinsed twice with chilled Dulbecco's calcium- and magnesium-free, phosphate-buffered saline (PBS) (Dainippon Pharmaceutical, Osaka, Japan). The number of viable chondrocytes was counted by using a Burker-Turk hemocytometer (Erma, Tokyo, Japan) with Trypan blue staining. Finally, the chondrocytes were seeded in 500 cm^2 ^square dishes (245 mm × 245 mm; Corning, Corning, NY, USA) at a density of 10,000 cells·cm^-2 ^and cultured in BM with 10% FBS at 37°C in an incubator with 5% CO_2_.

### Retroviral transfection

Retroviral transfection of cultured chondrocytes was performed as described previously [17,18] with some modifications. First, DH5α *Escherichia coli *cells (from Hiroshima University Graduate School of Biomedical Sciences) were cultured overnight in lysogeny broth (LB) media [19] at 37°C. Subsequently, these cells were transfected with p*hTERT*-MSCV and p*GRP78*-MSCV plasmids to produce amphotropic viruses. The plasmids were amplified and purified by using an EndoFree Plasmid Maxi Kit (Qiagen, Tokyo, Japan). In addition, the sequence of all constructs was verified by DNA sequencing.

Next, these plasmids were used to produce retroviral constructs by using 2 different protocols. To produce the *hTERT *retroviral construct, full-length *hTERT *cDNA was polymerase chain reaction (PCR) amplified, and then cloned into the pMSCV-puro retroviral vector (Clontech, Mountain View, CA, USA). Subsequently, the cloned vector was transfected into the Retropack PT67 (Clontech) packaging cell line and the transfected cells were selected with puromycin (1.8 μg·mL^-1^) (Sigma Aldrich, St. Louis, MO, USA) after 48 h. Two weeks after transfection, the surviving cells were trypsinized and allowed to continue to grow for up to 100 d. The culture supernatant from this cell line was collected and 0.45-μm filtered, and then polybrene (8 μg·mL^-1^) was added prior to transducing *hTERT *into young rabbit (YRA) and old rabbit (ORA) chondrocyte cultures.

Chondrocytes were cultured and plated 24 h before viral infection. Then, the packaged retrovirus was added to the culture media and incubated at 37°C in an incubator with 5% CO_2_. The infected cells were selected with 0.5 μg·mL^-1 ^of puromycin (Sigma Chemical) for 7-10 d prior to subsequent experiments.

To produce the *GRP78 *retroviral construct, full-length *GRP78 *cDNA was PCR amplified, and then inserted into the mouse stem cell virus (MSCV) packaging vector by using the Retrovirus Packaging Kit Ampho (TaKaRa Biotechnology, Shiga City, Japan) [20]. To produce *GRP78*-expressing retroviruses, 293 T cells (CRL-11268™, ATCC, Manassas, VA, USA) were seeded and maintained on 6-cm dishes at a density of 400,000 cells·cm^-2 ^in DMEM containing 10% FBS for 24 h prior to transfection. Then, the culture medium was changed to the same medium. Subsequently, the 293 T cells were co-transfected with pGP (gag-pol) and pE-ampho (env) (TaKaRa Biotechnology) by using calcium phosphate transfection. Transfected cells were selected with 400 μg·mL^-1 ^of hygromycin (Calbiochem, La Jolla, CA, USA) and 50 μg of mycophenolic acid (Sigma Aldrich). After 48 h, the culture medium, which contained the recombinant retroviruses, was 0.45-μm filtered, and then mixed with DMEM to infect YRA and ORA chondrocyte cultures.

### Chondrocyte proliferation

Chondrocyte proliferation was measured by counting cell numbers at 100% confluence in serial passages. Briefly, nontransfected or *hTERT*/*GRP78*-transfected YRA and ORA chondrocytes, which were passaged once every 7-10 d, were detached by using 0.05% trypsin/ethylenediaminetetraacetic acid (EDTA; Gibco) for 20-30 min at 37°C and washed 3 times with PBS. An aliquot of the detached cells was used to count the mean number of cells from 6 dishes by using a Burker-Turk hemocytometer (Erma) with Trypan blue staining. The remaining cells were replated at a density of 5 × 10^3 ^cells·well^-1^.

Cell proliferation was expressed as the population doubling level (PDL). The PDL was calculated from log-phase growth curves by using the equation: PDL = log_10 _(N/N_0_) × 3.33, where N_0 _and N are the number of cells at the beginning and end of each experiment, respectively [21].

### Preparation of the atelocollagen honeycomb-shaped scaffold with a membrane seal and 3-dimensional culture of chondrocytes

The ACHMS scaffold was prepared as described previously [13] by Koken (Tokyo, Japan). Briefly, nontransfected and *hTERT*/*GRP78*-transfected ORA chondrocytes were passaged twice, and then seeded at a density of 2 × 10^6 ^cells·scaffold^-1 ^into a round ACHMS scaffold (diameter, 6 mm; thickness, 2 mm; average pore size, 200 μm) [13,14,22,23] in 48-well plates (Sumitomo Bakelite, Tokyo, Japan) by centrifuging at 45 *g *for 5 min. Then, these cell-seeded scaffolds were cultured in BM supplemented with 10% FBS at 37°C in an incubator with 5% CO_2 _and 100% relative humidity for 14 d. These cultured chondrocytes were frozen in liquid nitrogen until needed for biochemical analyses and transplantation into an in vivo model of articular cartilage defects.

### Measurement of DNA and glycosaminoglycans

The amount of DNA in the cultured ORA chondrocytes, which was used as a marker of cell proliferation, was measured by digesting cell-seeded scaffolds with papain, and then using a fluorimetric assay, as described previously [24]. Briefly, 15 μL of a papain digest was mixed with 300 μL of Hoechst 33258 solution (Polyscience, Warrington, PA, USA), and then a Titertek Multiscan Spectrofluorometer (Lab Systems, Helsinki, Finland) was used to measure the emission and excitation spectra at 456 nm and 365 nm, respectively. DNA concentrations were calculated from a standard curve of calf thymus DNA (Sigma).

The amount of glycosaminoglycans (GAGs) in the cultured chondrocytes, which was used as a marker of extracellular matrix (ECM) formation, was quantified by using 1,9-dimethylmethylene blue, as described previously [25]. Briefly, samples (140 μL) of each chondrocyte culture were mixed gently with an equal volume of 1,9-dimethylmethylene blue solution in a 96-well microtiter plate, and then the absorbance at 530 nm was measured with a Titertec multiscan spectrophotometer (Labsystem, Helsinki, Finland). The amount of GAGs was calculated from the absorbance values by using a standard curve of 0.625-20 μg·mL^-1 ^shark chondroitin sulfate C (Seikagaku Kogyo Co, Tokyo, Japan).

### Type II collagen mRNA expression

Frozen 3-dimensional cultures of ORA chondrocytes were pulverized with a Cryo-Press (Microtec Nition, Chiba, Japan) in liquid nitrogen. All oligonucleotide primer sets were designed on the basis of published mRNA sequences. The expected amplicon lengths ranged from 70 to 200 bp. Cloning of the entire coding region of type 2 collagen was performed by 5'- and 3'-rapid amplification of cDNA ends using the following oligonucleotide primers: forward (5'-AACACTGCCAACGTCCAGAT-3'), reverse (5'-CTGCAGCACGGTATAGGTGA-3'). Real-time PCR was performed in a SmartCycler system (Cepheid, Sunnyvale, CA) with SYBR Green PCR Master Mix (Applied Biosystems, Foster City, CA) with 1 μL of cDNA template in a final volume of 25 μL. Amplification of cDNA was performed according to the following conditions: 95°C for 15 s and 60°C for 60 s for 35-45 amplification cycles. Changes in the fluorescence of SYBR Green were monitored after every cycle. Melting curve analysis was performed through a 0.5°C/s increase from 55 to 95°C with continuous fluorescence readings at the end of the cycles to ensure that single PCR products were obtained. All reactions were repeated in six separate PCR runs using RNA isolated from four sets of human samples. The results were evaluated by using SmartCycler software (Cepheid). Glyceraldehyde-3-phosphate dehydrogenase (GAPDH) primers were used to normalize the samples. To monitor crossover contaminations of PCR, RNase-free water (Qiagen, Valencia, CA) was used in the RNA extraction and as a negative control. To ensure the quality of data, a negative control was always included in each run.

### Implantation of tissue-engineered cartilage produced from transfected aged chondrocytes

Twenty four old female Japanese white rabbits were divided into 4 groups (control 8w, 16w; hTERT + GRP78 8w, 16w) and anesthetized with intramuscular injections of 120 mg of ketamine (Daiichisankyo, Tokyo, Japan) and 9 mg of xylazine (Bayer HealthCare, Leverkusen, Germany). After creating a medial parapatellar incision in both legs, each patella was dislocated laterally and a cylindrical defect (diameter, 5 mm; depth, 3 mm) was created on the patellar groove of the femur in both legs by using a biopsy punch (Kai Industries, Seki, Japan) and a low-speed drill (Takagi, Niigata, Japan). The bottom of the subchondral bone also was shaved to a plane until marrow bleeding was observed. Then, ACHMS scaffolds that were seeded with either nontransfected or *hTERT*/*GRP78*-transfected ORA chondrocytes were allografted into these defects without any fixatives, such as fibrin glue. Postoperatively, all animals were allowed to walk freely in their cages without any splints.

### Postoperative analyses

Eight and 16 weeks after implantation, rabbits were killed with an overdose of intravenous anesthesia, and then the distal parts of their femurs were harvested and observed with a light microscope. Subsequently, the femur samples were fixed in 10% buffered formalin for 7 d. Each specimen was decalcified with 10% EDTA in distilled water (pH 7.4) for 3 weeks, and then embedded in paraffin, cut into 6-μm-thick sagittal sections, deparaffinized, and stained with safranin O (Cartilage Staining Kit, Takara, Shiga, Japan). The histopathology of the OA cartilage samples (*n *= 24) were analyzed according to standard grading and staging of OA cartilage histopathology [26]. The OA score was calculated by the following formula: OA score = most degenerated site in the cartilage (grades 1-6, Table 1) × area of degeneration (stages 1-4) (Table 2).

**Table 1:** OA cartilage histopathology grade assessment; grading methodology

Grade (key feature)	Associated criteria (tissue reaction)
Grade 1: surface intact	Matrix: superficial zone intact, oedema and/or superficial fibrillation (abrasion), focal superficial matrix condensation Cells: death, proliferation (clusters), hypertrophy, superficial zone Reaction must be more than superficial fibrillation only
Grade 2: surface discontinuity	As above + Matrix discontinuity at superficial zone (deep fibrillation) ± Cationic stain matrix depletion (Safranin O or Toluidine Blue) upper 1/3 of cartilage ± Focal perichondronal increased stain (mid zone) ± Disorientation of chondron columns Cells: death, proliferation (clusters), hypertrophy
Grade 3: vertical fissures (clefts)	As above Matrix vertical fissures into mid zone, branched fissures ± Cationic stain depletion (Safranin O or Toluidine Blue) into lower 2/3 of cartilage (deep zone) ± New collagen formation (polarized light microscopy, Picro Sirius Red stain) Cells: death, regeneration (clusters), hypertrophy, cartilage domains adjacent to fissures
Grade 4: erosion	Cartilage matrix loss: delamination of superficial layer, mid layer cyst formation Excavation: matrix loss superficial layer and mid zone
Grade 5: denudation	Surface: sclerotic bone or reparative tissue including fibrocartilage within denuded surface. Microfracture with repair limited to bone surface
Grade 6: deformation	Bone remodelling (more than osteophyte formation only). Includes: microfracturewith fibrocartilaginous and osseous repair extending above the previous surface

Grade = depth progression into cartilage

**Table 2:** OA score; semi-quantitative method

Grade (key feature)	Stage % Involvement (surface, area, volume)			
		
	Stage 1 < 10%	Stage 2 10-25%	Stage 3 25-50%	Stage 4 > 50%
Grade 1(surface intact)	1	2	3	4

Grade 2 (surface discontinuity)	2	4	6	8

Grade 3 (vertical fissures, clefts)	3	6	9	12

Grade 4 (erosion)	4	8	12	16

Grade 5(denudation)	5	10	15	20

Grade 6(deformation)	6	12	18	24

Score = grade × stage

Immunohistochemical staining for type II collagen was performed as described previously [14]. Briefly, after deparaffinization, the sections were pretreated with 0.1 mg·mL^-1 ^of actinase E (Kaken Pharmaceutical) in PBS at 37°C for 30 min. Then, the sections were incubated with 10% pig serum at room temperature for 30 min to reduce nonspecific background staining. These pretreated sections were incubated overnight with 50 mg·mL^-1 ^mouse anti-human type II collagen monoclonal antibody (Daiichi Fine Chemical, Toyama, Japan) in PBS containing 0.1% bovine serum albumin at 4°C. Next, the sections were incubated with biotinylated rabbit anti-mouse immunoglobulin (1:500 dilution; Dako, Carpinteria, CA, USA) for 30 min at room temperature, followed by peroxidase-conjugated streptavidin (1:500 dilution; Dako) for 30 min at room temperature. Finally, the sections were incubated with a solution of 20 mg of diaminobenzidine and 5 μL of hydrogen peroxide (30%) in 100 mL of PBS for 5 min at room temperature. Control sections were incubated with PBS without any antibodies and stained in a similar manner. These sections were analyzed by light microscopy.

### Statistical analysis

One-way analysis of variance and Dunn's post hoc test was used to determine statistical significance (*P *< 0.05).

## Results

### Establishment of primary cultures of rabbit chondrocytes

**Figure 1:**
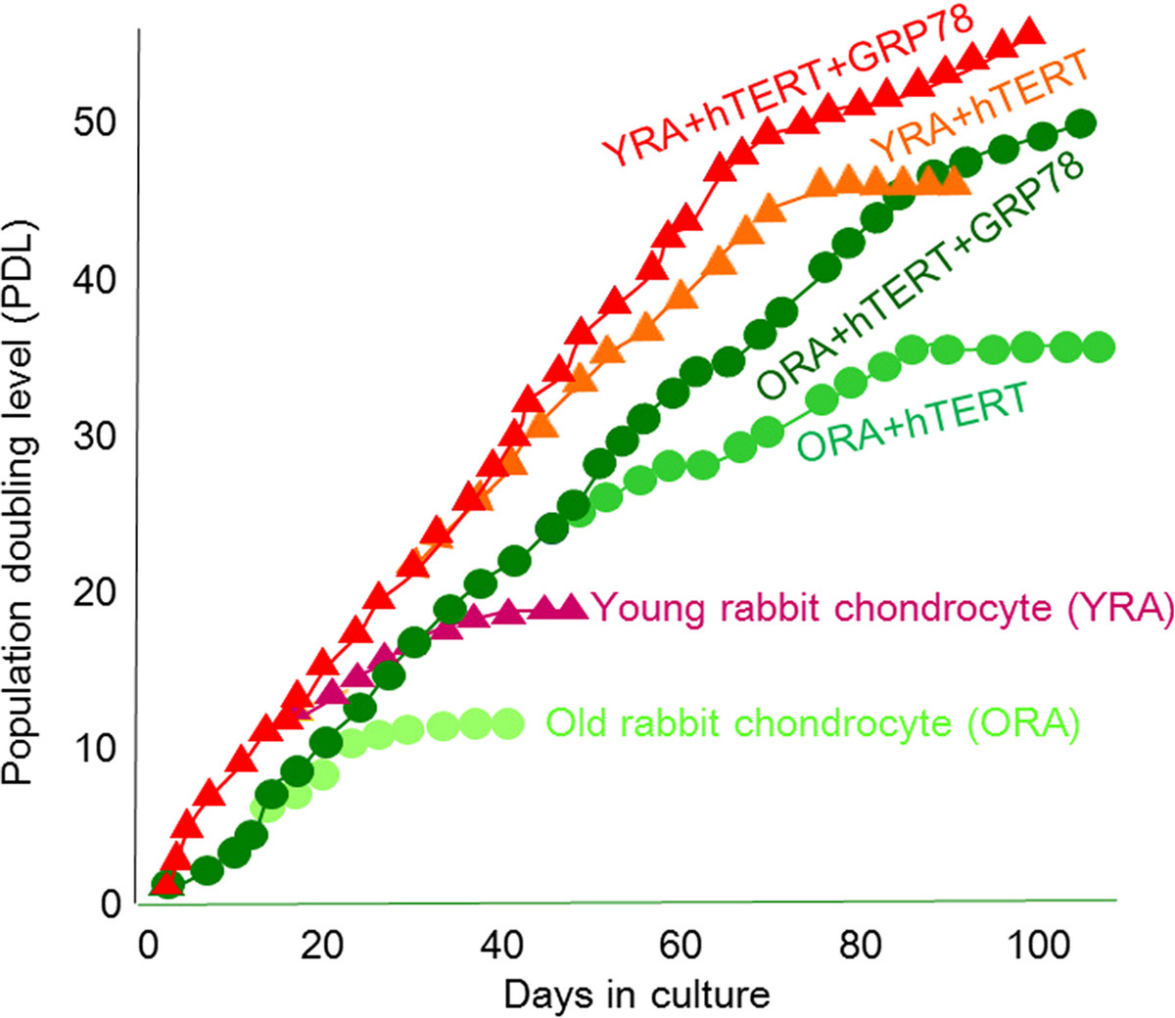
**Effect of *hTERT *and *GRP78 *on the proliferation of rabbit chondrocytes**. Growth curves of cultures of nontransfected control chondrocytes from young and old rabbits (YRA and ORA, respectively) and *hTERT *(YRA + *hTERT *and ORA + *hTERT*) and *GRP78 *(YRA + *hTERT *+ *GRP78 *and ORA + *hTERT *+ *GRP78*)-transfected chondrocytes. The mean number of cells is shown on a log_10 _scale.

During the first 3 weeks, YRA, ORA, ORA + *hTERT*, and ORA + *hTERT + GRP78 *chondrocytes showed similar growth rates for 10 PDL (Figure 1). Later, ORA + *hTERT *and ORA + *hTERT + GRP78 *chondrocytes proliferated more rapidly than the nontransfected chondrocytes. However, YRA + *hTERT + GRP78 *chondrocytes had the fastest growth rate. In the control groups, YRA chondrocytes proliferated faster than ORA chondrocytes during the entire observation period, but their growth rate gradually decreased until they ceased at about 40 d and 60 d after the initiation of culture, respectively. Unlike control cells, which stopped proliferating after 10-20 PDL, ORA + *hTERT *and YRA + *hTERT *cells continued proliferating for about 35 and 50 PDL, respectively. These results showed that *hTERT *and *GRP78 *increase the growth rate of transfected cells approximately 3-fold compared with nontransfected cells.

### Characteristics of the 3-dimensional cultures of chondrocytes

The ACHMS scaffold supported a high density of ORA chondrocytes (2 × 10^6 ^cells·cm^-2^) without any leakage of cells. During the 2-week culture, the chondrocytes in the scaffold retained their normal spherical shape (data not shown) and the resulting tissue-engineered cartilage maintained its shape and size in the ACHMS scaffold. The scaffolds were elastic and did not deform during culturing or collapse when handled with forceps.

**Figure 2:**
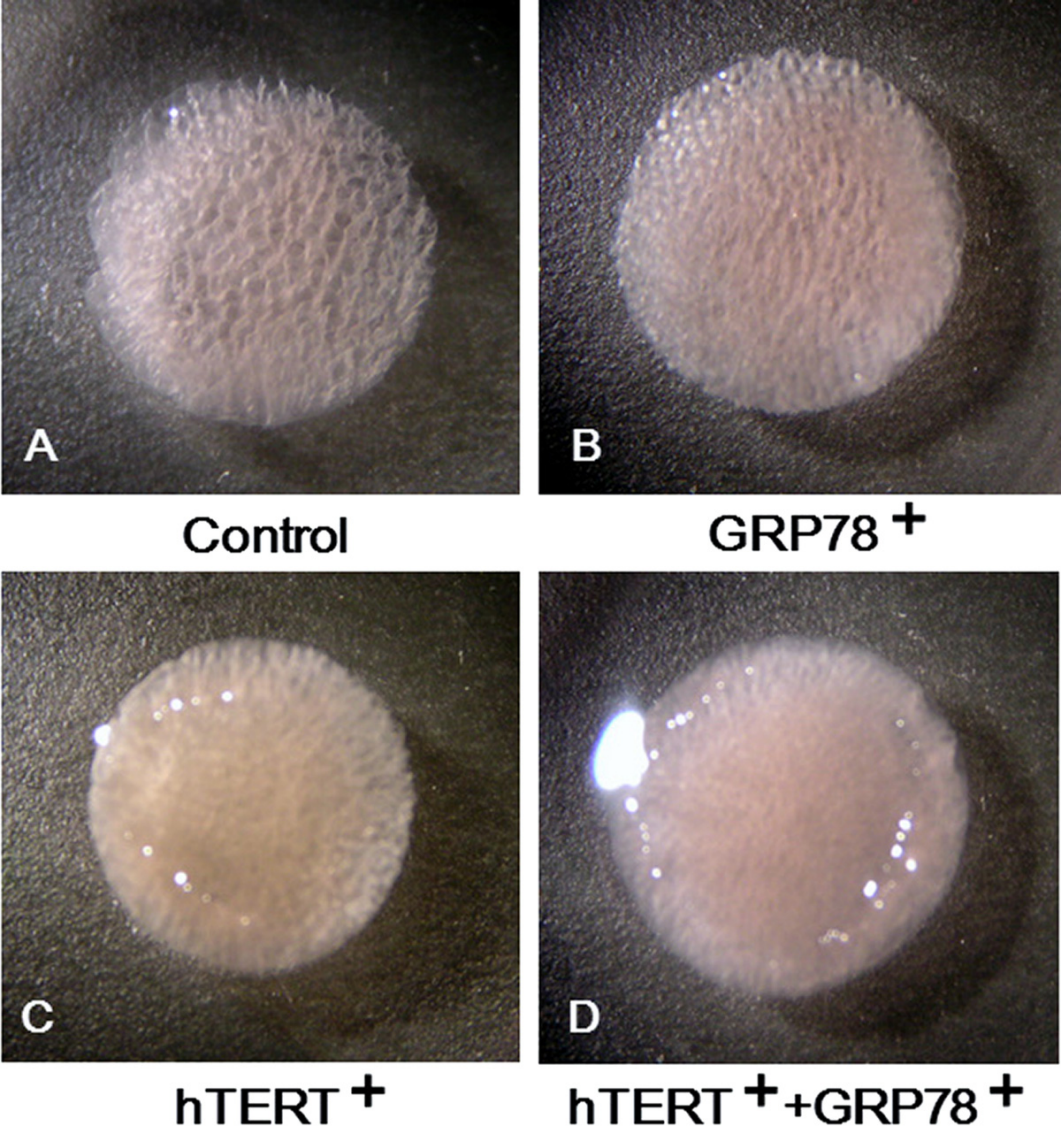
**Macroscopic images of the cell-scaffold complex after 14 days of culture**. Atelocollagen honeycomb-shaped scaffold with a membrane seal (ACHMS) scaffold complex seeded with nontransfected (A), *GRP78*-transfected (B), *hTERT*-transfected (C), or *hTERT*- and *GRP78*-transfected (D) ORA chondrocytes. Scale bar = 1.0 mm.

Figure 2 shows macroscopic images of the cell-seeded scaffolds after culturing for 14 d. The scaffold that was seeded with *hTERT*/*GRP78*-transfected ORA chondrocytes had the highest cell density. In addition, the spaces between the atelocollagen matrix were filled and not visible along the edge of the ACHMS scaffold, which indicated that chondrocytes had proliferated throughout the scaffold during the cultivation period. In the scaffolds that were seeded with control cells, cell growth was sparse, and as a result, the spaces between the atelocollagen matrix remained mostly empty.

### Glycosaminoglycan content of cell-seeded scaffolds

**Figure 3:**
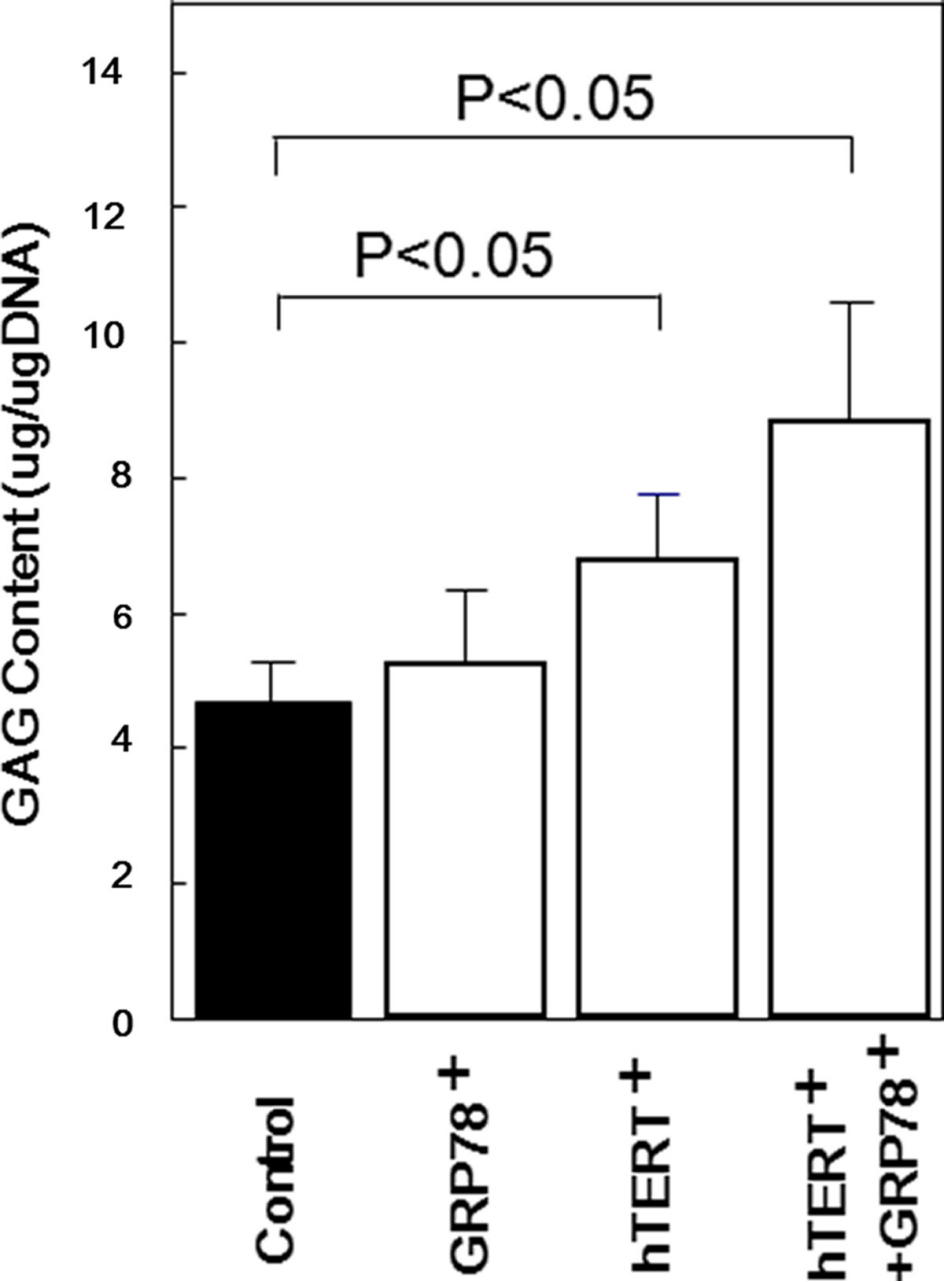
**Glycosaminoglycan content of chondrocyte cultures in atelocollagen honeycomb-shaped scaffolds with a membrane seal**. The glycosaminoglycan (GAG) content of nontransfected (black bars) or *hTERT*- or *GRP78*-transfected (white bars) ORA chondrocytes on the indicated days. Results are expressed as the mean (SD) of 6 independent measurements. *P < 0.05.

On day 14, the amount of GAG in cell-seeded scaffolds differed significantly between each group (Figure 3). Specifically, the total GAG content of scaffolds that were seeded with *hTERT/GRP78*-transfected ORA chondrocytes was higher than those that were seeded with *GRP78*- or *hTERT*-transfected cells. In addition, the GAG content of the scaffolds that were seeded with transfected ORA chondrocytes was higher than that in those that were seeded with nontransfected chondrocytes. These results suggested that transfected ORA chondrocytes were able to produce and accumulate significantly higher amounts of extracellular matrix components in the ACHMS scaffold than nontransfected chondrocytes.

### Type II collagen mRNA expression

**Figure 4:**
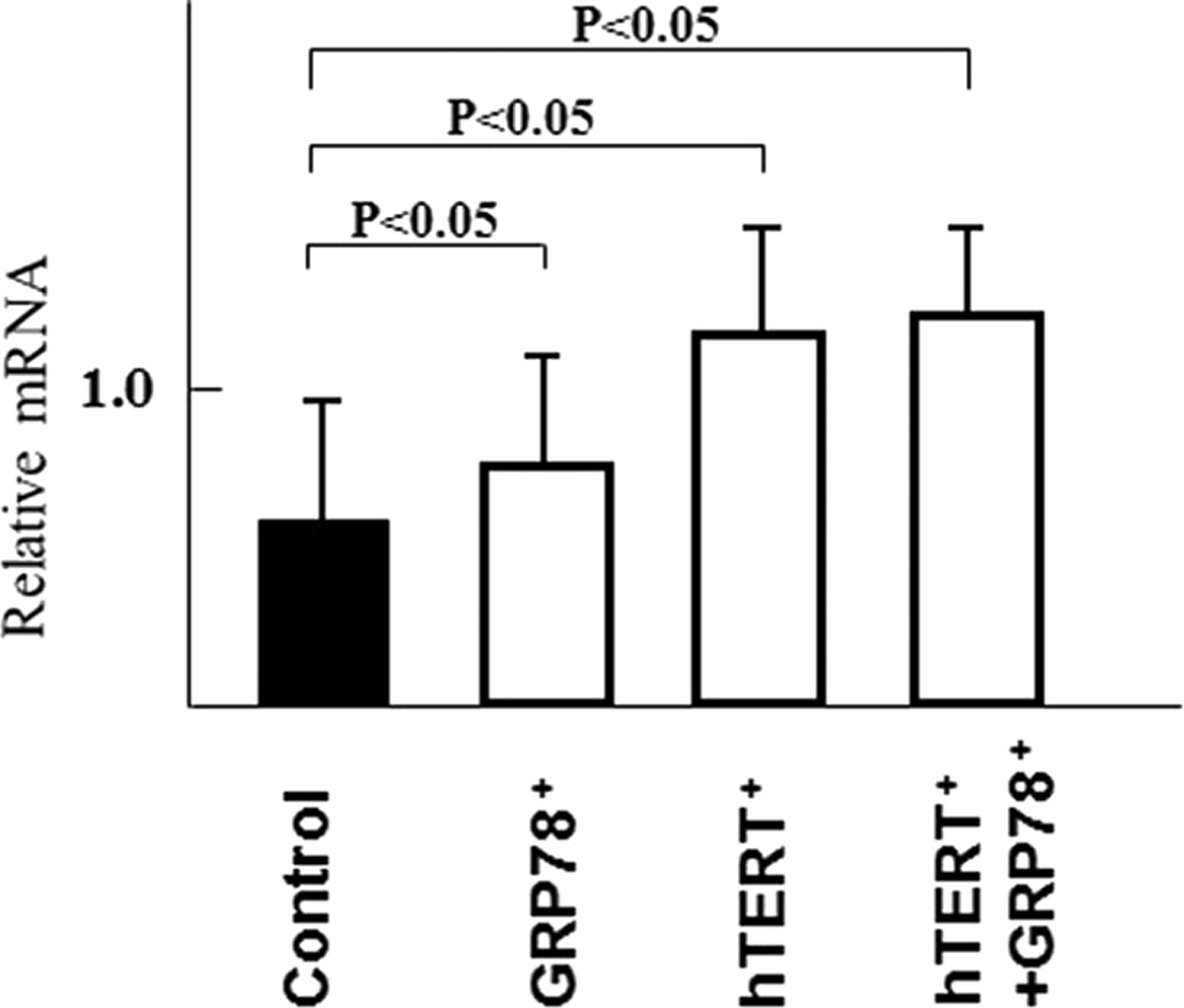
**mRNA expression levels of type II collagen in transfected chondrocytes**. The mRNA expression of type II collagen was observed in ORA, regardless of different gene transfection. Profiles of control, *GRP78*^+^, *hTERT*^+^, and *hTERT*^+^/*GRP78*^+ ^cells were prepared to investigate expression of type II collagen. The mRNA expressions of *GRP78*^+^, *hTERT*^+^, and *hTERT*^+^/*GRP78*^+ ^cells were significantly higher than that in control of non-transfective ORA. GAPDH was used as an internal control to normalize RNA levels.

As shown in Figure 4, the mRNA expression of type II collagen was observed in ORA, regardless of different gene transfection. Profiles of control, *GRP78*^+^, *hTERT*^+^, and *hTERT*^+^/*GRP78*^+ ^cells were prepared to investigate expression of type II collagen. The mRNA expressions of *GRP78*^+^, *hTERT*^+^, and *hTERT*^+^/*GRP78*^+ ^cells were significantly higher than that in control of non-transfective ORA. Chondrocytes are known to readily dedifferenciate in 2-dimensional culture. However, in this study, we used the primary culture of chondrocytes so that these chondrocytes retain their phenotype, which can be confirmed from type II collagen expression.

### Macroscopic appearance of repaired osteochondral defects

The surgical implantation of tissue-engineered cartilage into osteochondral defects in the old rabbits was uneventful, and upon waking, all rabbits immediately resumed normal cage activity. At the time that they were killed, all rabbits exhibited unlimited passive range of motion in the knee joint.

**Figure 5:**
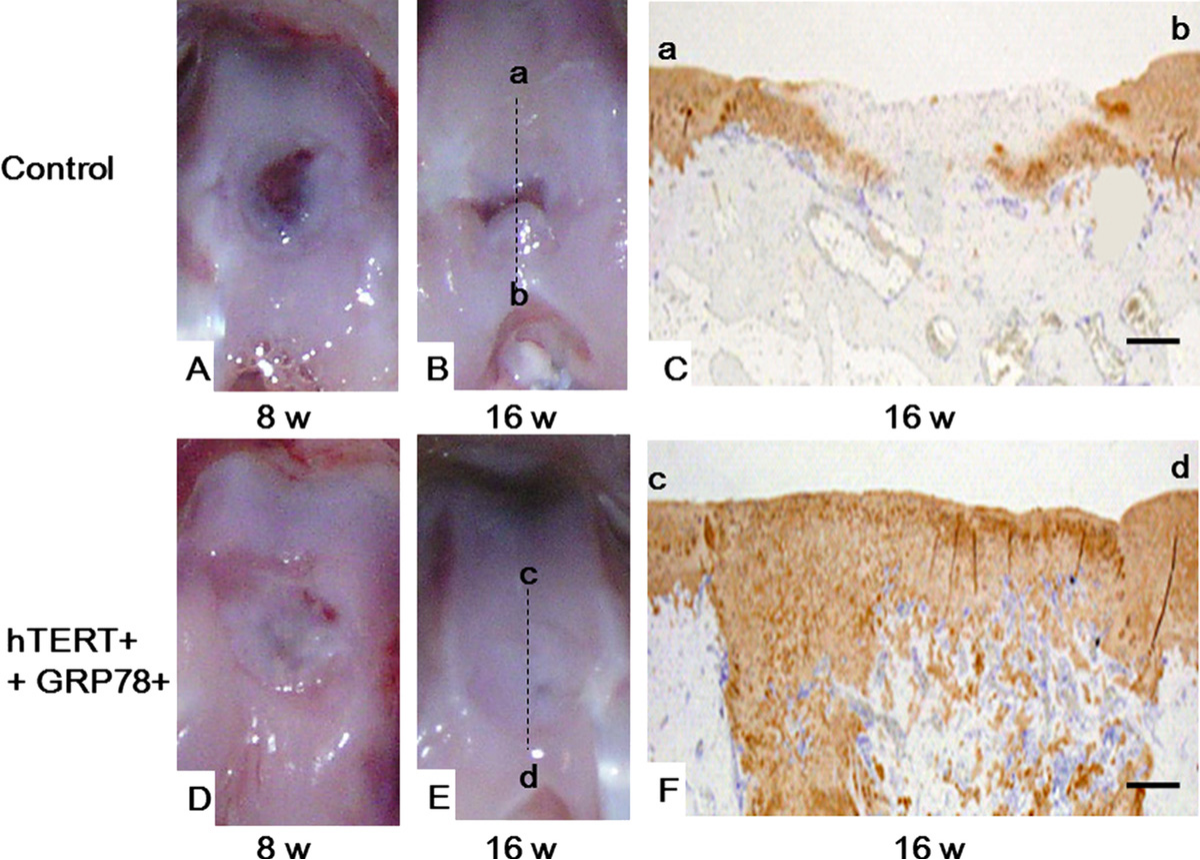
**Macroscopic and immunostaining of regenerated cartilage after implantation**. Postoperative appearance of repaired osteochondral defects in old rabbits implanted with scaffolds seeded with either nontransfected (top panels) or *hTERT*- and *GRP78*-transfected ORA chondrocytes (bottom panels) either 8 (A, D) or 16 (B, E) weeks after surgery. Immunostaining of type II collagen in tissue sections from rabbits implanted with scaffolds seeded with nontransfected (C) or *hTERT*- and *GRP78*-transfected (F) ORA chondrocytes. Each micrograph is representative of 8 tissue samples. Scale bar = 1.0 mm.

Indeed, the osteochondral defects in old rabbits that were treated with tissue-engineered cartilage that was grown from *hTERT*- and *GRP78*-transfected ORA chondrocytes were filled with smooth tissue that resembled hyaline cartilage 16 weeks after surgery (Figure 5E) unlike the tissue-engineered cartilage that was grown from nontransfected ORA chondrocytes, which remained empty or were covered by fibrous tissue (Figure 5B). Although the control tissue-engineered cartilage showed some tissue repair along the borders of the defect, the color of the tissue was slightly different from that of the surrounding normal cartilage (Figure 5A, B).

### Histological analysis of repaired osteochondral defects

No signs of arthritis, such as cartilage erosion or severe synovial proliferation, were observed in any surgically treated knee. In rabbits that were implanted with tissue-engineered cartilage grown from *hTERT*- and *GRP78*-transfected ORA chondrocytes, the immunohistochemical staining for type II collagen in the extracellular matrix in the scaffold was more intense and covered a larger area (Figure 5F) than those that were implanted with scaffolds that were grown from nontransfected ORA chondrocytes (Figure 5C). In addition, the tissue-engineered cartilage that was grown from transfected ORA chondrocytes was smooth and displayed good bonding with the host cartilage on both sides. In contrast, the tissue-engineered cartilage that was grown from nontransfected ORA chondrocytes had an irregular surface and was thinner than that grown from *hTERT*- and *GRP78*-transfected chondrocytes (Figure 5C).

**Figure 6:**
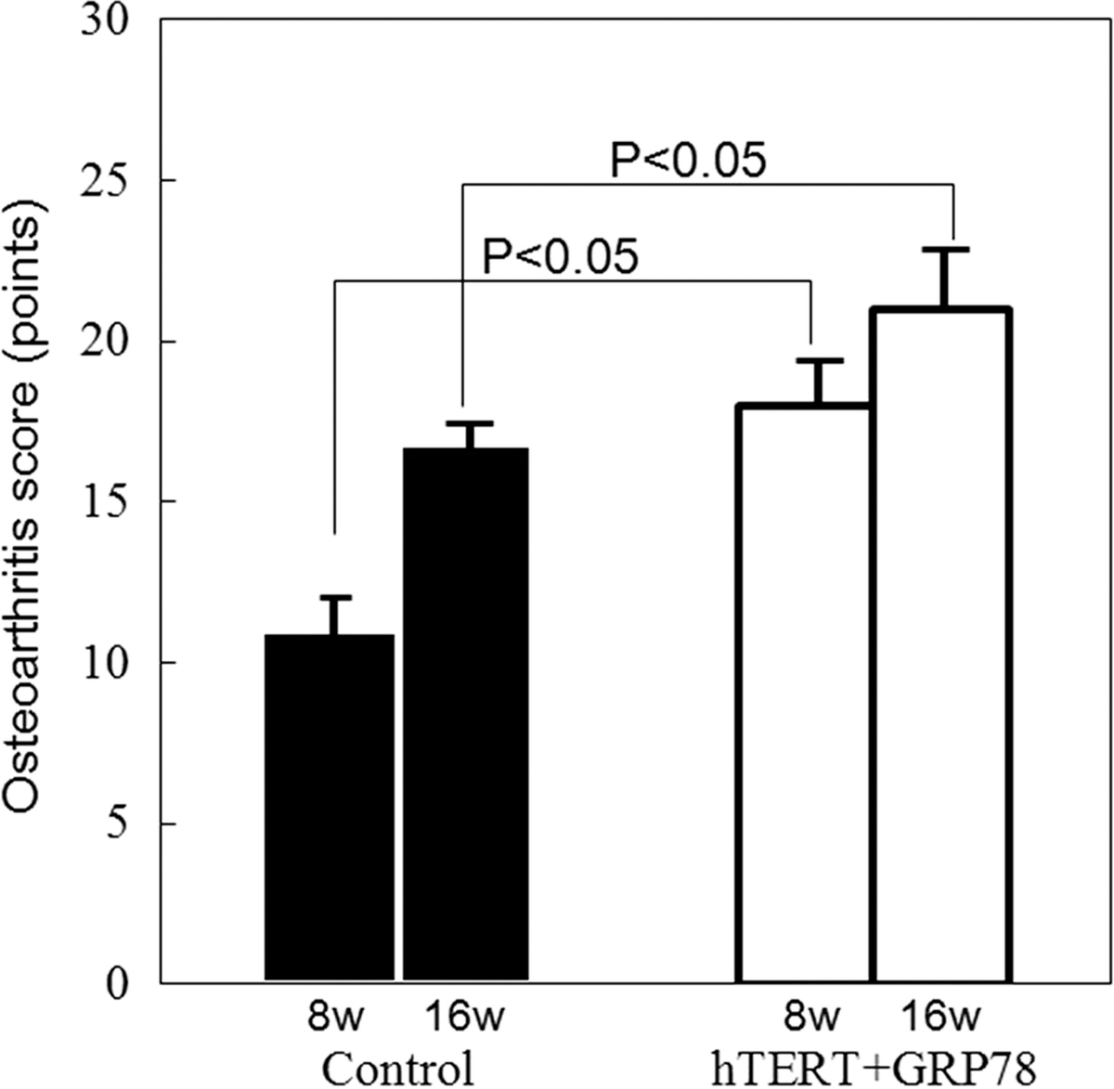
**Osteoarthritis score**. The histopathology of the OA cartilage samples from all 4 groups (*n *= 24) were analyzed according to standard grading and staging of OA cartilage histopathology. OA scores were calculated by using the following formula: OA score = most degenerated site in the cartilage (grades 1-6, Table 1) × area of degeneration (stages 1-4) (Table 2). Error bars indicate the standard deviation of each group. Statistically significant differences were observed between the control and hTERT + GRP78 groups 8 and 16 weeks after transplantation.

As shown in Figure 6, the average OA scores were as follows: control 8w, 11.2 (2.5); 16w, 16.0 (3.2); hTERT + GRP78 8w, 17.3 (3.5); 16w, 21.00 (2.85). Statistically significant differences (P < 0.05) were observed between the transfected and nontransfected groups that were observed for the same period of time after implantation.

## Discussion

Aging is the most important risk factor for both the initiation and progression of degenerative cartilage diseases. Osteoarthritic cartilage degeneration may be due to the loss of viable chondrocytes due to apoptosis or physical stress. This degeneration is likely to be closely related to age-related changes, since aging chondrocytes and articular cartilage matrix undergo senescence-like changes, which increase the susceptibility of cells to degenerative processes and environmental or physiological stresses [27]. As a result, chondrocytes from osteoarthritic patients might progress toward senescence more rapidly than those from normal individuals.

Aging chondrocytes also have important therapeutic ramifications. Recently, the treatment of articular cartilage defects have improved with the introduction of advanced tissue engineering techniques for autologous chondrocyte implantation (ACI) [28]. ACI requires cell expansion in culture to provide sufficient amounts of chondrocytes for reimplantation. However, like all mammalian cells, normal adult chondrocytes have a limited mitotic potential and eventually enter a state of senescence [29]. Moreover, the replicative life span of primary cells in culture are affected by the age of the donor, such that cells from older donors have a shorter life spans than those from younger donors [30]. For example, in monolayer cultures of aged human chondrocytes, serial passages rapidly results in loss of phenotypic stability and proliferative capacity [7]. Thus, to facilitate the therapeutic use of chondrocytes from older donors, a method is needed to prolong their replicative life span.

One possible method is transfection of *hTERT*, which can immortalize or prolong the life span of various human cells, such as muscle satellite cells [31,32], myoblasts [33,34], fibroblasts [6], and chondrocytes [7,8]. Since most immortalized cells maintain their phenotype and state of differentiation, *hTERT*-transfected cells are considered potential therapies for small-cell lung cancer [35] and for postnatal neovascularization in severe ischemic disease [36]. However, since chondrocytes uniquely maintain their phenotypes in 3-dimensional cultures [37], it is not known whether *hTERT*-immortalized chondrocytes maintain their state of differentiation.

Due to the aforementioned issues, in most cartilage tissue engineering studies, donor chondrocytes are usually from young animals. Our study is the first report that transfection of *hTERT *and *GRP78 *can increase the replicative life span and therapeutic potential of tissue-engineered cartilage that is produced from ORA chondrocytes, which have a limited regenerative capacity. In addition, we used an ACHMS scaffold to maintain the phenotype of transfected chondrocytes, as indicated by the production of GAGs and type II collagen (Figures 3 and 4). These results are consistent with the findings of Piera-Velazquez et al. [8].

In this study, we overcame the limited lifespan of ORA chondrocytes by transfection with *hTERT *and increased their growth rate up to 3-fold by cotransfection with *GRP78 *(Figure 1). Specifically, *hTERT/GRP78*-transfected ORA chondrocytes grew at a constant rate for more than 20 PDL, whereas nontransfected chondrocytes stopped dividing after much fewer PDL. However, we were not able to completely immortalize chondrocytes, even those from young rabbits (PDL < 50). Although the additional transfection of SV40-TAg or mutant Ras could immortalize these cells, we did not choose this option because the transfected cells may have become cancerous. As a result, we focused on the phenotypic stability of GRP78 and hTERT.

*hTERT *is a candidate gene for gene therapy of muscular dystrophy [31-34]. In contrast, *GRP78 *may have therapeutic applications for neuropathological conditions, such as Alzheimer's disease, because it protects cells from ER stress [11,38-40]. ER stress can alter protein synthesis in cells [41]. One mechanism by which ER stress promotes apoptosis in cells is by driving the accumulation of structurally abnormal proteins [42], which are ordinarily repaired by ER chaperones to prevent age-related cell death. GRP78 is an example of a chaperone protein that regulates protein folding in the ER and thus contributes to cell survival [43]. Since the increase in the expression of GRP78 during cell culture may help protect cells from ER stress, overexpression of GRP78 also may protect cultured chondrocytes independent of hTERT.

Due to a lack of cages for mutant rabbits, we were not able to perform animal transplantation experiments with chondrocytes that were transfected with *hTERT *or *GRP78 *alone. However, we believe that our in vitro and in vivo results from chondrocytes that were transfected with both *hTERT *and *GRP78 *are sufficient to support our conclusions. In the future, we plan to perform more animal experiments to elucidate the effects of *GRP78*.

In conclusion, our results showed that tissue-engineered cartilage that was grown from implanted *in vivo *with *hTERT*- and *GRP78*-transfected ORA chondrocytes in ACHMS scaffolds can repair articular cartilage defects in vivo (Figure 5D, E, F). The *hTERT *and *GRP78*-transfected ORA exhibited proliferative and differentiation activity in articular cartilage defects, resulting in the formation of hyaline cartilage. This study also shows that ORA chondrocytes potentially produce hyaline cartilage after genetic treatment, similar to chondrocytes from young animals. However, the mechanical strength of regenerated articular cartilage in large animals (*i.e*., sheep or pigs) needs to be investigated.

## Abbreviations

hTERT: Human telomerase reverse transcriptase; GRP78: Glucose-regulated protein 78; ER: Endoplasmic reticulum; ACHMS scaffold: Atelocollagen honeycomb-shaped scaffold with a membrane seal; OA: Osteoarthritis; BM: Basal medium; DMEM: Dulbecco's modified Eagle's medium; FBS: Fetal bovine serum; YRA: Young rabbit; ORA: Old rabbit; PDL: Population doubling level; RT-PCR: Reverse transcriptase-polymerase chain reaction; GAPDH: Gylceraldehyde-3-phosphate dehydrogenase; ACI: Autologous chondrocyte implantation; ECM: Extracellular matrix.

## Competing interests

The authors declare that they have no competing interests.

## Authors' contributions

MS, KS, MI, and TN conducted the experiments. MS, MI, NK, TK, HT, and GM analyzed the data. JIL performed the statistical analyses. MS, JIL, and JM wrote the manuscript. All authors read and approved the final manuscript.

## Pre-publication history

The pre-publication history for this paper can be accessed here:

http://www.biomedcentral.com/1471-2474/13/51/prepub
